# Ultralow Threshold Lasing from Carbon Dot–Ormosil Gel Hybrid-Based Planar Microcavity

**DOI:** 10.3390/nano11071762

**Published:** 2021-07-06

**Authors:** Yiqun Ni, Zhixia Han, Junkai Ren, Zhen Wang, Wenfei Zhang, Zheng Xie, Yonghong Shao, Shuyun Zhou

**Affiliations:** 1 Key Laboratory of Optoelectronic Devices and Systems of Ministry of Education and Guangdong Province, College of Physics and Optoelectronic Engineering, Shenzhen University, Shenzhen 518060, China; 2160190410@email.szu.edu.cn; 2 Key Laboratory of Photochemical Conversion and Optoelectronic Materials, Technical Institute of Physics and Chemistry, Chinese Academy of Sciences, Beijing 100190, China; hanzhixia15@mails.ucas.ac.cn (Z.H.); renjkfs@163.com (J.R.); wangzhen.wza@hotmail.com (Z.W.); zhou_shuyun@mail.ipc.ac.cn (S.Z.); 3 University of Chinese Academy of Sciences, Beijing 100049, China; 4 Shenzhen Key Laboratory of Laser Engineering, College of Physics and Optoelectronic Engineering, Shenzhen University, Shenzhen 518060, China

**Keywords:** carbon dots, ormosil gel hybrids, ultralow threshold lasing, planar microcavity

## Abstract

The absence of an ideal solid matrix with resistance to harsh conditions for carbon dots (CDs) and high transmittance in the visible/near infrared region is the bottleneck in CD applications. In this study, we show that a stable rigid structure can be formed between CDs and organically modified silicates (ormosil) gel when CDs are incorporated into ormosil gel hybrids as a solid matrix. A high photoluminescence quantum yield (PLQY) of 63% is achieved at a 583 nm emission. Peak optical gain of the hybrids was found to be 67 cm^−1^ at peak wavelength. Ultralow threshold (~70 W/cm^2^) lasing can also be demonstrated from a planar microcavity by using CD–ormosil gel hybrids as a gain medium.

## 1. Introduction

Recently, fluorescence nanomaterials have been applied in a number of technologies such as biosensing, photoelectric devices, light emitting diodes, lasers, multiplexed imaging, and so forth because of their excellent luminescent properties [1,2,3,4,5]. Among them, carbon dots (CDs) have attracted increasing interest as novel fluorescent nanomaterials due to their outstanding properties, such as great biocompatibility, upconversion photoluminescence (UCPL), high quantum yields (QYs), excellent photostability, easy surface functionalization, and environmental sustainability [6,7,8,9]. Thus, CDs have already been regarded as one kind of potential alternative for traditional semiconductor materials in various technologies, including photoelectric devices and sensors photocatalysis, lasing emission, etc. [7,10,11,12,13]. Typically, these applications usually require materials in solid states owing to significant stability and practical applications. However, most CDs suffer severe fluorescence quenching in the solid aggregate state and even show no fluorescence, which limits the application of CDs significantly [14,15,16].

To overcome fluorescence quenching, much effort has been devoted to developing an ideal solid matrix that includes natural polymers and synthetic macromolecules [13,16,17]. Among them, types of polymers such as poly (methyl methacrylate) (PMMA), polystyrene (PS), and polyvinyl alcohol (PVA) are usually selected as typical solid matrixes. PMMA is a favorite polymeric matrix due to its high transparency in the visible region and excellent mechanical properties [13]; polystyrene is frequently used as a thin film matrix because of its optically clear thermoplastic and flexible properties [18]; polyvinyl alcohol is taken into account since its hydroxyl groups can act as sources of hydrogen bonding [19]. Comparing with these common solid materials, sol-gel glasses derived from organically modified silicates (ormosil) have attracted increasing attention for embedding functional dopants in various optical materials and devices due to their excellent performance, including simple preparation process, adequate resistance to harsh conditions (high temperature, corrosion, etc.), high transmittance in the visible (Vis) and near infrared (NIR) region, long-term stability, as well as excellent mechanical properties [20,21]. 

A suitable matrix with low light absorption, long-term stability, and rigid structure are also crucial to CD lasers. The first CD-based laser was based on a liquid matrix, and then the solid CD-based laser using a CD/epoxy composite as a gain medium achieved a low threshold random lasing of ~200 W/cm^2^ in the green region by our group [17,22]. Afterward, the CD-based lasers with different emission colors (ranging from ultraviolet to red light) in both the liquid and solid states were reported widely by different researchers [7,8,23,24,25,26]. However, there are two main drawbacks, hindering the CD lasers in practical applications. Firstly, most CD lasers suffer from a high lasing threshold (the majority of them are about several kW cm^−2^) [7]. Secondly, the liquid characteristics of most CDs also restrict laser device fabrication. A solid laser cavity with low light loss can be beneficial to practical applications of CD lasers [17]. Therefore, further improvement of the optical and physical properties in a solid matrix of CDs is required for practical CD laser applications.

**Figure 1:**
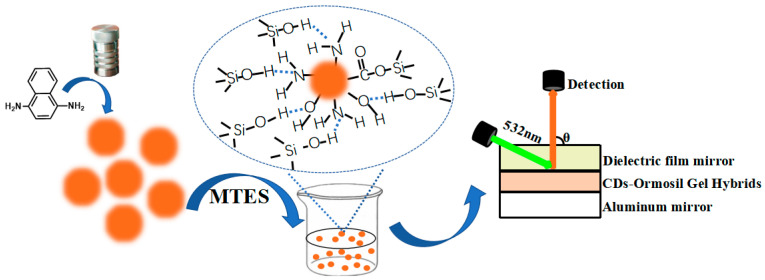
Schematic diagram showing the preparation of carbon dot (CD)-gel and planar microcavity laser.

In this work, we developed a CD-gel by using ormosil gel as a solid matrix, which is an ideal matrix for CDs due to its high resistance to harsh conditions and high transmittance. More importantly, its rigid molecular structure, which is beneficial to stabilize the surface states of CDs and suppress non-radiative recombination, is able to facilitate a high photoluminescence quantum yield [20,27,28]. A maximum QY of 63% was achieved from the CD-gel at 583 nm. Then planar microcavity lasers with the as prepared hybrids as a gain medium could be fabricated by sandwiching the CD-gel film between an aluminum film mirror and a dielectric mirror (Figure 1), showing that the orange laser emission with an ultralow threshold of ~70 W/cm^2^ can be achieved. To the best of our knowledge, the lasing threshold is the lowest value achieved and orders of magnitude lower than that of most of other CD lasers ever recorded in previous publications.

## 2. Results and Discussion

### 2.1. Optical Properties of CD-gel

**Figure 2:**
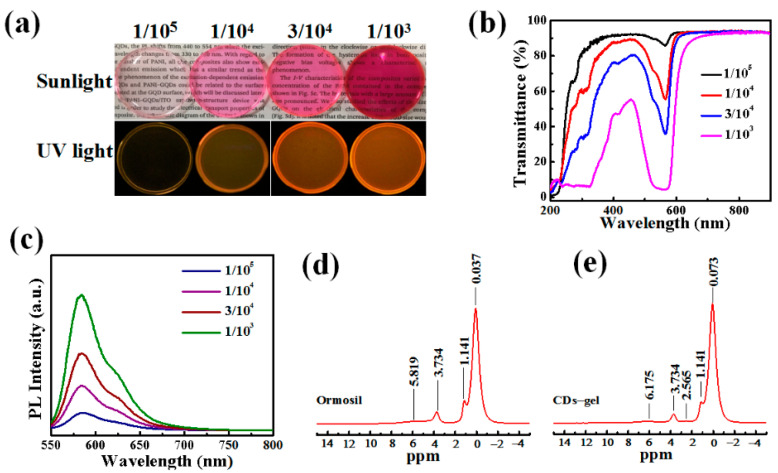
(**a**) Photography of CD-gel under sunlight and ultraviolet light; (**b**) transmittance spectra and (**c**) photoluminescence emission spectra under excitation of 532 nm of different mass concentrations of CDs to organically modified silicates (ormosil) gel; (**d**) the solid state nuclear magnetic resonance (SSNMR) of ormosil gel; (**e**) the SSNMR of CD-gel.

CDs were obtained via the simple one-step solvothermal method (see Figure 1) [7]. For applications in optical devices, the outstanding optical properties of CDs have been transformed from liquid state to solid state via employing samples into prepolymer of methyl triethoxysilane (MTES). These CD-gels are named 1/10^5^, 1/10^4^, 3/10^4^ and 1/10^3^ CD-gels, respectively. Here, 1/10^5^, 1/10^4^, 3/10^4^ and 1/10^3^ correspond to different mass concentrations of CDs to ormosil gels. The following optical measurements are preformed to explore the performance of CD-gels (see Figure 2). Figure 2a shows the optical photographs of the four CD-gels under sunlight and ultraviolet (UV) light, respectively. As shown in Figure 2b, obvious absorption peaks at 532 and 568 nm were observed from the transmittance spectra of the four CD-gel films, indicating no influences in absorption wavelength with difference concentration CD solutions. Furthermore, the 3/10^4^ CD-gel had moderate transmittance in the visible spectrum and high absorption at the 532 nm, which was the optimal concentration. Their photoluminescence emissions were observed with a peak at 583 nm (see in Figure 2c), which can be regarded as no having distinction with CD ethanol solution. Meanwhile, the full width at half maximum (FWHM) was obtained with a value of 45 nm, which is wider than CD ethanol solution [7]. To investigate the relationship between CDs and ormosil gel, solid state nuclear magnetic resonance (SSNMR, see in Figure 2d,e) was applied to ormosil gel and CD-gel. The hydrogen existence forms in ormosil gel are -CH_3_, -OCH_2_CH_3_ and Si-OH, whose chemical shift are 0.073, 1.414, 3.734 and 5.819 ppm, respectively. While in the CD-gel, the peak at δ = 2.565 appeared and the peak at the δ = 5.819 ppm moved to the low field at 6.175 ppm, which could have been caused by the hydrogen bond effect. There are -OH, -NH_2_ and -COOH molecules on the surface of CDs, according to our previous research [7]. Therefore, the new peak at δ = 2.565 vests in -NH_2_ in the CDs and the -COOH becomes an alicyclic group with Si-OH. The -OH and -NH_2_ form a hydrogen bond with Si-OH and the density of the electron cloud around Si-OH decreases. Consequently, the shielding effect on Si-OH decreases and the chemical shift moves to the low field. This may be also one of the reasons that the full width at half maximum was wider in the CD-gel.

**Figure 3:**
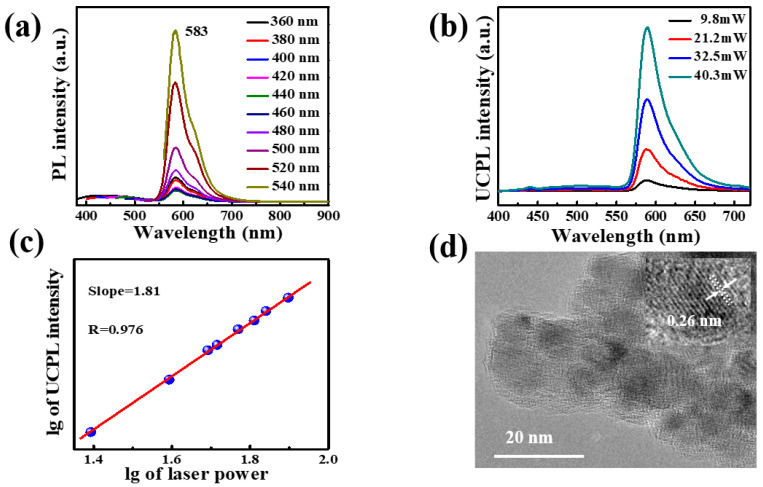
(**a**) Photoluminescence emission spectra under different excitation wavelengths; (**b**) upconversion photoluminescence spectra with different laser powers by a femtosecond pulse laser (λ_ex_ = 800 nm); (**c**) plot of upconversion photoluminescence intensity and laser power; (**d**) transmission electron microscopy (TEM) image (inset showing high-resolution TEM image) of 3/10^4^ CD-gel.

Due to an optimal optical property, the 3/10^4^ CD-gel was selected for further research. Figure 3a describes that the photoluminescence of 3/10^4^ CD-gel can be excited with a wide range of wavelengths and the emission peak position is virtually independent of the excitation wavelength. Furthermore, photoluminescence QY of the 3/10^4^ CD-gel was determined to be as high as 63%. The similar emissions between upconversion photoluminescence and one-photon photoluminescence (see Figure 3b,c) were also observed using an 800 nm femtosecond pulsed laser with a clear quadratic relationship between the excitation laser power and the luminescence intensity. The transmission electron microscopy (TEM) image (Figure 3d) revealed that the uniform-sized CDs were homogeneously dispersed in the ormosil gel film of 3/10^4^ CD-gels. The lattice spacing of CDs was 0.26 nm, corresponding to the (020) planes of graphene [11].

### 2.2. Ultralow Threshold Laser

**Figure 4:**
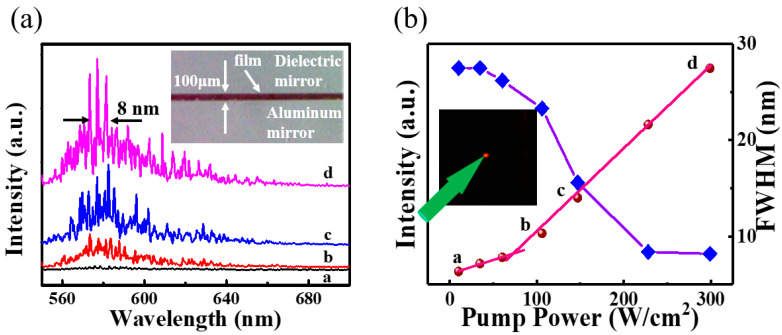
(**a**) Lasing spectra of CDs laser under different excitation powers (532 nm excitation). The inset shows a photograph of a cross-section of the laser cavity; (**b**) Output intensity and linewidth of the emission spectra versus pump power. The inset shows a photography of lasing emission from the CDs laser under 532 nm excitation.

Bestowed with superior optical properties such as high QYs and low light absorption, 3/10^4^ CD-gel is compelling for application in lasers. With CD-gel as optical gain media, a planar microcavity laser composed of two reflection mirrors and a thin CD-gel film was designed to achieve optical feedback and lasing emission [29]. As shown in the schematic diagram (Figure 1), the CD-gel film was sandwiched between an aluminum mirror and a dielectric film mirror to form a planar microcavity. The inset of Figure 4a shows a photograph of a cross-section of the microcavity taken by an optical microscope. It was observed that the CD-gel film was about 100 μm thick. The reflectivity of the aluminum film mirror and the dielectric film mirror were about 94% and 45%, respectively, with respect to the photoluminescence peak wavelength of the CD-gel between 560 and 600 nm (as shown in Figure S1). The purpose of using the two mirrors was to improve the longitudinal confinement of light inside the planar microcavity [8,29]. 

A 532-nm pulsed laser with a 6 ns pulse width was utilized as an excitation source. The fluorescent lifetime of the CDs was determined to be 10 ns (Table S1). As a result, it is possible to achieve population inversion in the CD-gel film. The 532-nm pulsed laser was focused onto a spot of 500 μm in diameter on the CD-gel film through a convex lens at room temperature. Light emission from the planar microcavity was coupled into a spectrometer via an optical fiber perpendicular to the mirror plane (i.e., θ = 90°) (see Figure 1). As shown in Figure 4a, when pump power was low, a small bump formed at about 570–605 nm in the spectra. This indicates that only spontaneous emission was observed. However, if the pump power further increased, sharper peaks of lasing modes emerged in the spectra. The inset of Figure 4b shows a photograph of a bright emitting light recorded by a camera. Meanwhile, full width at half maximum of the emission spectra reduced to 8 nm (see Figure 4b) with pump power increasing to ~300 W/cm^2^ and a kink at about 70 W/cm^2^ (i.e., threshold of the laser) was formed in the light-light curve in Figure 4b. This means that the spontaneous emission began to convert to the stimulated emission at this pump power. When the pump power was higher than 70 W/cm^2^, stimulated emission was dominant over the spontaneous radiation.

**Figure 5:**
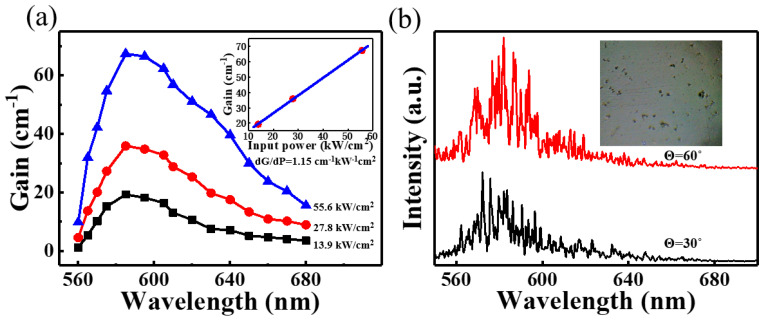
(**a**) Optical gain spectra of CD-gel film versus different excitation power densities. The inset shows the peak optical gain of CD-gel film versus excitation power densities; (**b**) Lasing spectra of the CD laser observed from different detection angles. The inset shows a photograph of the CD-gel film.

These results indicate that ultralow threshold lasing was achieved in our planar microcavity laser successfully. Comparisons of pump thresholds and QYs of different CD lasers in previous reports are summarized in Table S2. It is shown that the threshold of the CD-gel laser was found to be orders of magnitude lower than that of others CD lasers. This could be due to two reasons. Firstly, high optical gain, which can be benefited from the high photoluminescence efficiency of the CD-gel, is the key for ultralow threshold lasing [30]. Figure 5a plots the net optical gain spectra, which were measured by using the variable stripe length method [17,30], of the CD-gel under a 532 nm wavelength excitation. It was observed that the peak optical gain was about 67 cm^−1^ at peak wavelength under pump excitation at 55.6 kW/cm^2^. The inset of Figure 5a plots the peak optical gain of the CD-gel versus pump intensity. It was noted that the peak optical gain per pump power, which was determined to be 1.15 cm^−1^ kW^−1^ cm^2^, was much higher than that of previous reported CDs [17]. Secondly, this planar microcavity laser could provide twofold optical confinements. This is because the vertical resonances were determined by the planar microcavity. Simultaneously, the microscale thin film gain medium provided extra optical confinement (laser modes can only resonate in the film plane) [8,29]. As a result, the planar microcavity laser could provide twofold optical confinements, and the threshold of the laser could be reduced drastically. 

It is shown in Figure 4a that the spacing of laser modes were randomly and unevenly distributed in the emission spectra, which were different from classic Fabry–Perot lasers. This could be due to inhomogeneous distribution of refractive indices in the CD-gel film. The inset of Figure 5b shows a photograph of the CD-gel film taken by an optical microscope. It can be observed that microscale craters (~10 μm in diameter), which were generated by the evaporation of small molecules (i.e., gas molecules) during solidification, were formed on the surface of the CD-gel film. The inhomogeneous surface caused the inhomogeneous distribution of refractive indices inside the CD-gel film, leading to the random scattering of light [8]. The random lasing phenomenon can be verified by lasing spectra detected from different emission angles. Figure 5b shows the emission spectra detected at different emission angles (θ = 60° and θ = 30°). It can be observed that different lasing spectra could be detected from different angles and all the lasing modes were randomly distributed. These results indicate that the proposed microcavity laser performs characteristics of random lasers with the CD-gel film as a gain media.

## 3. Conclusions

In summary, a carbon dot–ormosil gel hybrid with a maximum photoluminescence QY of 63% at 583 nm were developed by using ormosil gel as a solid matrix. Peak optical gain of the CD-gel was about 67 cm^−^^1^ at peak wavelength under 532 nm excitation at 55.6 kW/cm^2^. Furthermore, a solid-state planar microcavity laser was fabricated by sandwiching a layer of CD-gel gain medium film between an aluminum film mirror and a dielectric film mirror. Ultralow-threshold lasing (~70 W/cm^2^) was orders of magnitude lower than that of most CD lasers in previous reports. This was a result of the superior optical characteristics of our CD-gel as well as twofold optical confinement provided by the planar microcavity. This demonstration indicates that CDs are a high-efficiency luminescent material with excellent optical characteristics and may have potential to be used as a laser gain medium in a low-threshold diode pump laser.

## 4. Experimental Section

### 4.1. Synthesis of CD-gel

0.05 g 1,4-diaminonaphthalene was ultrasonically dissolved in 20 mL ethanol to form a transparent solution. The solution was then transferred into autoclaves and placed in an oven for heating at 200 °C for 12 h. After cooling to room temperature, the obtained suspensions were purified via silica column chromatography using dichloromethane and methanol as the eluent. Finally, the CDs solution was obtained after evaporation of the eluent. Fifty milliliters MTES was added in 100 mL ethanol and the transparent solution was mixed well. Thirteen and a half milliliters water of pH = 2.5 (adjusted by HCl) was divided into three parts and one part was added into the transparent solution every half hour. The transparent solution was then stirred for 12 h and suspension steamed to 75 mL. The transparent solution was then divided into different parts and put into the molds and different concentrations of CDs were added to produce mass concentrations of 1/10^5^, 1/10^4^, 3/10^4^ and 1/10^3^ CD-gel.

### 4.2. Fabrication of the Laser Cavity

The Fabry–Perot (FP) laser cavity was fabricated by spin coating a layer of 3/10^4^ CDs with ormosil gel on an aluminum mirror at 1000 rpm for 10 s, the CD layer was then cured into CD-gel by heating at 80 °C for 10 min, and finally a dielectric film mirror was covered on the surface of the hybrid film to form the FP laser cavity. 

### 4.3. Characterization Methods

A JEM-2100F instrument (JEOL, Tokyo, Japan) and Multimode 8 in strument (Bruker, Madison, WI, USA) were used to investigate the morphologies of CDs, which included taking TEM and AFM images. An Excalibur HE 3100 (Varian, Palo Alto, CA, USA) and ESCALab250i-XL electron spectrometer (Thermo Fisher Scientific, Waltham, MA, USA) were used to record the chemical constitutions, such as FT-IR and XPS, of CDs. Fluorescence spectra, UV−vis absorption, and transmittance spectra were measured on an F-4500 instrument (Hitachi, Tokyo, Japan) and U-3000 instrument (Hitachi, Tokyo, Japan). The resolved fluorescence lifetime measurement was recorded using an LP920 instrument (Edinburgh instruments, Edinburgh, UK). Absolute QYs were obtained using an integrating sphere connected by a FLS980 system (Edinburgh Instruments, Edinburgh, UK). ^1^H solid-state NMR measurements were carried out by 600 MHz JNM-ECZ600R JEOL RESONANCE Inc instrument (Edinburgh Instruments, Edinburgh, UK). NMR spectrometers equipped with 1.0 mm triple resonance and double resonance ultrafast MAS probes, respectively.

Lasing characteristics of CDs were studied via third harmonic generation from a YAG pulsed laser (355 nm, 10 Hz) with an optical parameter oscillator to expand the YAG laser to different excitation wavelengths. The 532 nm laser beam was focused onto a spot on the CD film with diameter about 500 μm through a lens. The light emitted from the laser cavity was detected by a monochromator via an optical fiber. 

## Data Availability

Data is contained within the article or Supplementary Materials.

## Supplementary Materials

The following are available online at https://www.mdpi.com/article/10.3390/nano11071762/s1, Figure S1: Reflectivity of the Al film mirror and the dielectric film mirror; Table S1: PL decay lifetimes τ and the relative fluorescence intensity percentages f for CDs ethanol solution, and χ^2^ is the reduced Chi-Square value for τ_avg_; Table S2: Pump threshold, QYs, excitation wavelength and emission wavelength of different CD lasers in recent reports.
