# Supplementary material for: Ultralow Threshold Lasing from Carbon Dot–Ormosil Gel Hybrid-Based Planar Microcavity
Source: Nanomaterials (Basel). 2021 Jul 6;11(7):1762. doi: 10.3390/nano11071762 (PMC8307971; doi:10.3390/nano11071762)
Supplement: Supplementary file 1 [file nanomaterials-11-01762-s001.zip › nanomaterials-1270267-supplementary.pdf]

Ultralow Threshold Lasing from Carbon Dot–Ormosil Gel Hybrid-Based Planar Microcavity

Yiqun Ni [1,†], Zhixia Han [2,3,†], Junkai Ren [2,3], Zhen Wang [2,3], Wenfei Zhang [1,4,*], Zheng Xie [2,*], Yonghong Shao [1,*] and Shuyun Zhou [2,3]

1 Key Laboratory of Optoelectronic Devices and Systems of Ministry of Education and Guangdong Province, College of Physics and Optoelectronic Engineering, Shenzhen University, Shenzhen 518060, China; 2160190410@email.szu.edu.cn (Y.N.)
2 Key Laboratory of Photochemical Conversion and Optoelectronic Materials, Technical Institute of Physics and Chemistry, Chinese Academy of Sciences, Beijing 100190, China; hanzhixia15@mails.ucas.ac.cn (Z.H.); renjkfs@163.com (J.R.); wangzhen.wza@hotmail.com (Z.W.); zhou_shuyun@mail.ipc.ac.cn (S.Z.)
3 University of Chinese Academy of Sciences, Beijing 100049, China
4 Shenzhen Key Laboratory of Laser Engineering, College of Physics and Optoelectronic Engineering, Shenzhen University, Shenzhen 518060, China
* Correspondence: zhangwf@szu.edu.cn (W.Z.); zhengxie@mail.ipc.ac.cn (Z.X.); shaoyh@szu.edu.cn (Y.S.)
† These authors contributed equally in this work.

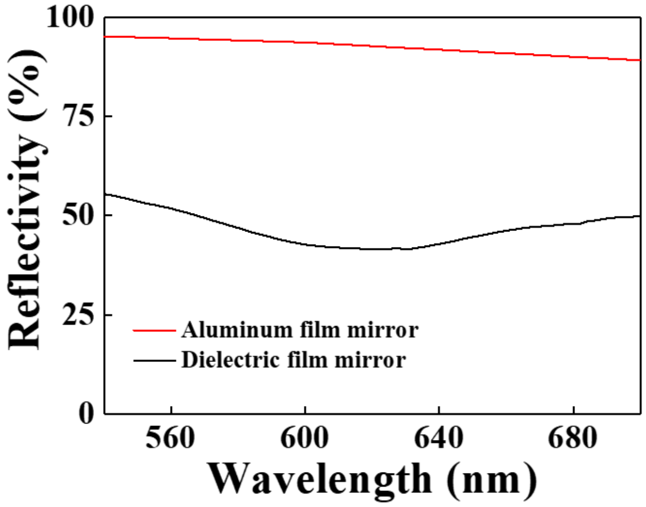

Figure S1. Reflectivity of the Al film mirror and the dielectric film mirror.

Table S1. PL decay lifetimes τ and the relative fluorescence intensity percentages f for CDs ethanol solution, and χ^2 is the reduced Chi-Square value for τ_{avg}.

Sample	τ_1	f_1	τ_2	f_2	τ_{avg}	χ^2
CDs	2.129 ns	5.36%	10.452 ns	94.64%	10.0 ns	1.164

Table S2. Pump threshold, QYs, excitation wavelength and emission wavelength of different CD lasers in recent reports.

Type	Pump threshold ($kW\ cm^{-2}$)	QY (%)	λ_{ex} (nm)	λ_{em} (nm)	References
CDs/epoxy composite	0.2	68	450	556	[1]
CDs	80	82	360	458	[2]
Graphene QDs	40	-	266	375	[3]
carbon-nanoparticles	200	36	355	555	[4]
CDs-Ormosil gel hybrids (this work)	0.07	63	532	581	

References

1. Zhang, W.; Jin, L.; Yu. S. Zhu, H.; Pan, S.; Zhao, Y.; Yang, H. Wide-bandwidth lasing from C-dot/epoxy nanocomposite Fabry-Perot cavities with ultralow threshold. *J MATER CHEM C* **2014**, *2*, 1525–1531.
2. Zhang, Y.; Hu, Y.; Lin, J.; Fan, Y.; Li, Y.; Lv, Y.; Liu, X. Excitation Wavelength Independence: Toward Low-Threshold Amplified Spontaneous Emission from Carbon Nanodots. *ACS APPL MATER INTER* **2016**, *8*, 25454–25460.
3. Zhu, H.; Zhang, W.; Yu, S. Realization of lasing emission from graphene quantum dots using titanium dioxide nanoparticles as light scatterers. *NANOSCALE* **2013**, *5*, 1797–1802.
4. Qu, S.; Liu, X.; Guo, X.; Chu, M.; Zhang, L.; Shen, D. Amplified Spontaneous Green Emission and Lasing Emission From Carbon Nanoparticles. *ADV FUNCT MATER* **2014**, *24*, 2689–2695.
